# *Brucella suis* S2 strain inhibits IRE1/caspase-12/caspase-3 pathway-mediated apoptosis of microglia HMC3 by affecting the ubiquitination of CALR

**DOI:** 10.1128/msphere.00941-24

**Published:** 2025-02-28

**Authors:** Zhao Wang, Yanbai Wang, Shulong Yang, Zhenhai Wang, Qian Yang

**Affiliations:** 1 Department of Experimental Surgery, The Second Affiliated Hospital of Air Force Medical University, Xi'an, China; 2 Department of Neurology, The General Hospital of Ningxia Medical University, Yinchuan, China; 3 Department of Orthopedics, The People’s Hospital of Wuhai, Wuhai, China; 4 Diagnosis and Treatment Engineering Technology Research Center of Nervous System Diseases of Ningxia Hui Autonomous Region, The General Hospital of Ningxia Medical University, Yinchuan, China; Washington University in St. Louis School of Medicine, St. Louis, Missouri, USA

**Keywords:** neurobrucellosis, *Brucella suis *S2, endoplasmic reticulum stress, microglia, apoptosis, IRE1/caspase-12/caspase-3 pathway

## Abstract

Neurobrucellosis represents a severe complication of brucellosis, posing a considerable risk to human health and quality of life. This condition arises from an increased susceptibility to chronic *Brucella* infection, a significant clinical challenge. One key factor contributing to chronic neurobrucellosis is the regulation of microglial apoptosis by *Brucella*; however, the exact molecular mechanisms remain largely unresolved. In this study, human microglial clone 3 (HMC3) cells were infected with *Brucella suis* vaccine strain S2 (*B. suis* S2) at varying multiplicity of infection (MOI) and durations to assess its effects on the IRE1/caspase-12/caspase-3 signaling pathway. Following the suppression of this pathway by *B. suis* S2, calreticulin (CALR) was identified through ubiquitin-modified proteomics (data accessible via ProteomeXchange, identifier PXD056006). To further investigate, CALR-overexpression and knockdown HMC3 cell lines were infected with *B. suis* S2 to elucidate the mechanism by which *B. suis* S2 inhibits apoptosis in HMC3 cells. In conclusion, our findings demonstrate that *B. suis* S2 suppresses HMC3 cell apoptosis via the IRE1/caspase-12/caspase-3 pathway by modulating CALR ubiquitination. This study provides a theoretical basis for exploring the mechanisms of neurobrucellosis and offers insights into its clinical treatment.

**IMPORTANCE:**

Neurobrucellosis is a severe complication impacting the central nervous system (CNS) due to neurological deficits caused by *Brucella*, with primary clinical features including meningitis, encephalitis, brain abscesses, and demyelinating lesions. These nonspecific symptoms often lead to misdiagnosis or delayed diagnosis, increasing the risk of recurrent or chronic neurobrucellosis infections. Consequently, persistent infection and relapse are critical challenges in the clinical management of neurobrucellosis, which are closely linked to *Brucella*’s survival and replication within microglia. Interestingly, *Brucella* may inhibit microglia apoptosis by mitigating endoplasmic reticulum (ER) stress, though the precise molecular mechanisms remain largely unexplored. Thus, this study will elucidate the specific mechanisms by which *Brucella* suppresses microglial apoptosis and provide deeper insights into the molecular pathogenesis and clinical treatment of neurobrucellosis.

## INTRODUCTION

Brucellosis is a prevalent zoonotic disease worldwide, affecting multiple organ systems (1) with broad, nonspecific clinical manifestations (2). Among these, neurobrucellosis is a severe complication impacting the central nervous system (CNS) due to neurological deficits caused by *Brucella* (3). Approximately 30% of brucellosis cases eventually progress to neurobrucellosis (4), with primary clinical features including meningitis, encephalitis, brain abscesses, and demyelinating lesions (5). These nonspecific symptoms often lead to misdiagnosis or delayed diagnosis, increasing the risk of recurrent or chronic neurobrucellosis infections (6). Most patients endure long-term neurological dysfunction and disability, imposing a significant medical burden on families and society (7). Consequently, persistent infection and relapse are critical challenges in the clinical management of neurobrucellosis.

The persistent infection and recurrence of neurobrucellosis are closely linked to *Brucella’*s survival and replication within microglia (8). As the CNS’s resident macrophages, microglia play a vital role in identifying and eliminating invading pathogens (9). Once inside macrophages, *Brucella* forms *Brucella*-containing vacuoles (BCVs), which are membrane-bound compartments that facilitate bacterial survival (10). BCVs then localize to the endoplasmic reticulum (ER) and acquire ER-derived membranes (11), allowing *Brucella* to replicate and regulate host cell apoptosis (12). The ER serves as the primary site for intracellular Ca^2+^ storage, as well as protein synthesis and folding (13). As a result, bacteria can trigger ER stress in host cells by causing an accumulation of misfolded or unfolded proteins (14). Interestingly, while *Brucella* can induce ER stress, studies have shown that it may also inhibit macrophage apoptosis by mitigating ER stress, though the precise molecular mechanisms remain largely unexplored (15). Thus, the persistent infection and relapse of neurobrucellosis may be closely related to the regulation of ER stress-associated apoptosis in microglia by *Brucella*. Unraveling the underlying molecular mechanisms regulating this process would be beneficial for reducing relapse and persistent infection of neurobrucellosis.

Host cells detect ER stress through ER-resident transmembrane proteins such as inositol-requiring enzyme 1 (IRE1), activating transcription factor 6 (ATF6), and protein kinase RNA-like ER kinase (PERK) (16). Of these, IRE1 is the most conserved and has been extensively studied regarding its role in regulating ER stress-related apoptosis in response to bacterial infections (14, 17). Although *Brucella suis* has been shown to inhibit apoptosis in goat trophoblast cells by activating IRE1 to promote intracellular replication (14), other research indicates that *Brucella abortus* suppresses IRE1 to alleviate ER stress and reduce trophoblast cell death (18). The exact mechanisms through which IRE1 regulates apoptosis remain unclear. Caspase-12, an ER stress response caspase localized within the ER (19), is a key mediator of ERS-associated apoptosis (20). Upon ER stress, pro-caspase-12 is activated and subsequently activates caspase-3, leading to apoptosis (21). However, *Brucella*’s role in modulating microglial apoptosis via the IRE1/caspase-12/caspase-3 pathway has not yet been reported in the literature, which is the central focus of our study.

Pathogenic bacteria exploit host cell protein quality control pathways to regulate not only ER stress (22) but also protein ubiquitination (23). Ubiquitination, a complex enzymatic process, attaches ubiquitin to substrate proteins, marking them for degradation to ensure proper stability, localization, and activity (24). By modulating host ubiquitination, intracellular bacteria can promote their survival (25). For instance, *Helicobacter pylori* is known to persist in gastric mucosa by manipulating the host ubiquitin system (26). Disruptions in protein ubiquitination lead to the buildup of unfolded or misfolded proteins, which may aggravate ER stress (27). For *Brucella*, to ensure survival and proliferation within the host cell, they can regulate lysosomal membrane permeability- and autophagy-related apoptosis (28, 29). However, reports on Brucella regulating ER stress-related apoptosis in microglia by altering host ubiquitination are scarce.

This study first established the optimal treatment duration and multiplicity of infection (MOI) for *Brucella suis* vaccine strain S2 (*B. suis* S2) to inhibit the IRE1/caspase-12/caspase-3 pathway in human microglial clone 3 (HMC3) cells. Subsequently, calreticulin (CALR), a protein linked to both apoptosis and ER function, was identified through quantitative ubiquitin-based proteomics (ubiquitomics). CALR is a calcium-binding chaperone protein predominantly localized in the ER (30) and is upregulated under ER stress to maintain ER homeostasis (31). Further investigation focused on how *B. suis* S2 modulates CALR levels to influence apoptosis in HMC3 cells. This research aims to elucidate the specific mechanisms by which *B. suis* S2 suppresses microglial apoptosis and to provide deeper insights into the molecular pathogenesis of neurobrucellosis.

## MATERIALS AND METHODS

### Kits, antibodies, and lentivirus

Whole Protein Extraction Kit (lot#: 20191011), BCA Protein Quantification Kit (lot#: 20191210), and SDS-PAGE Reagent Kit (lot#: 20190327) were sourced from KeyGen Biotech Co. Ltd. (Nanjing, China). The RNA simple Total RNA Kit (lot#: S8108) was provided by Tiangen Biotech (Beijing, China), while the Transcriptor First-Strand cDNA Synthesis Kit (lot#: 00823782) was obtained from Thermo Fisher Scientific (Waltham, USA). Vazyme Biotech (Nanjing, China) supplied the ChamQ SYBR qPCR Master Mix (lot#: 7E371J9). Endoplasmic Reticulum Staining Kit (lot#: BB-441164) and Intracellular Calcium Staining Kit (lot#: BB-44129) were purchased from Bestbio Biotechnology (Shanghai, China). The Annexin V-APC/7-AAD Apoptosis Kit (lot#: HA0716) was obtained from US Everbright (Suzhou, China). Primary antibodies for rabbit anti-p-IRE1 (Ser724, lot#: GR271918-30), anti-cleaved-caspase-3 (lot#: GR297363-10), anti-cleaved-caspase-12 (lot#: GR3306653-9), anti-GAPDH (lot#: GR200347-16), and anti-calreticulin (lot#: GR3252549-6) were procured from Abcam (Cambridge, UK). Antibodies against IRE1 (lot#: I09262562) were provided by Wanlei Biotechnology (Shenyang, China). IRDye 800CW goat anti-rabbit IgG (H+L) (lot#: C81210-05) was supplied by Li-COR (Lincoln, NE, USA). CALR-ZsGreen and sh-CALR-ZsGreen lentiviruses were acquired from Hanbio Biotechnology (Shanghai, China).

### Cell culture

The HMC3 cell line was purchased from Kelei Biotechnology (Shanghai, China) and routinely tested to ensure they were mycoplasma-free, with authentication through STR detection. Cells were cultured in high-glucose Dulbecco’s modified Eagle medium (DMEM; Gibco, Shanghai, China) supplemented with 10% fetal bovine serum (FBS; Gibco, Grand Island, NY, USA) and 1% penicillin/streptomycin/amphotericin B. They were maintained in an incubator at 37°C with 5% CO_2_. When cultures in 10 cm dishes or 25-cm^2^ flasks reached approximately 80% confluence, cells were washed twice with phosphate-buffered saline (PBS; Hyclone, Shanghai, China) and passaged at a 1:4 ratio.

### Bacteria and bacterial culture

*B. suis* S2 was supplied by the Ningxia Key Laboratory of Clinical Pathogenic Microorganisms (biosafety level 2; Yinchuan, China). This strain is a live attenuated vaccine and is widely used to study the *Brucella* virulence and the mechanism of brucellosis (32, 33). The lyophilized *B. suis* S2 was stored at −80°C and resuspended in tryptic soy broth (TSB; Hope Biotechnology, Qingdao, China), followed by dilution and inoculation onto tryptone soya agar (TSA; Hope Biotechnology, Qingdao, China) plates. Cultures were incubated for 4 days at 35°C in 5% CO_2_. A single colony was selected and cultured in 10 mL of TSB at 37°C, shaking at 180 rpm for 36 hours. Bacterial cells were then centrifuged at 4,500 rpm for 5 minutes, washed twice with PBS, and bacterial concentrations were determined using a turbidimetric method for further experiments. All procedures involving *B. suis* S2 were conducted in a BSL-2 laboratory, adhering to strict biosafety protocols.

### *In vitro* experiments by bacterial infection

A drop of bacterial droplet was cast on a slide, and the water was removed using an alcohol lamp flame. Subsequently, the purity of the *Brucella* solution was determined through Kozlovsky staining. HMC3 cells were seeded at a density of 2 × 10^6^ cells per 10 cm dish in an antibiotic-free complete medium. Upon reaching 60% confluence, cells were infected with *B. suis* S2 at varying multiplicities of infection (MOI is defined as the quantity ratio of bacteria with host cells; 25, 50, 100, and 200) for different time intervals (1, 2, 4, and 8 hours). After infection, cells were washed three times with PBS to eliminate extracellular bacteria and harvested for subsequent analysis.

### Western blot analysis

The HMC3 cell density was maintained as previously described. When cell confluency exceeded 90%, Western blot analysis was conducted to assess the protein levels of IRE1/p-IRE1, cleaved caspase-12, and cleaved caspase-3. Cells from each group were digested with 0.25% trypsin, neutralized with a complete medium, and harvested by centrifugation at 1,000 rpm for 5 minutes. Total proteins were extracted using the Whole Protein Extraction Kit, following the manufacturer’s protocol. After centrifugation at 14,000 rpm for 30 minutes at 4°C, protein concentrations were quantified using a BCA Protein Quantification Kit. Protein lysates (80 µg per group) were mixed with 5× loading buffer and denatured by heating in a 100°C metal bath for 8 minutes. Proteins were then separated on 8%–12% SDS-PAGE gels and transferred onto 0.45 µm polyvinylidene difluoride (PVDF) membranes. Non-specific binding sites on the PVDF membranes were blocked by incubation with 5% (wt/vol) skimmed milk in Tris-buffered saline containing 0.1% Tween (TBST) for 1.5 hours at room temperature. Membranes were incubated overnight at 4°C with primary antibodies (1:500–1:1,000), followed by five 5 minute washes with TBST. Subsequently, the membranes were incubated with IRDye 800CW Goat anti-Rabbit IgG (H+L) at 37°C for 1 hour. Protein visualization was performed using the Odyssey Clx Infrared Imaging System (LI-COR, Lincoln, USA). The mean optical densities (MOD) of the target proteins were measured and normalized to GAPDH for relative protein expression.

### Endoplasmic reticulum staining

For immunofluorescence staining, HMC3 cells were seeded at a density of 1.5 × 10^5^ cells per well on coverslips in 12-well plates. Upon reaching 60% confluency, the cells were infected with *B. suis* S2. Endoplasmic reticulum staining was carried out according to the Endoplasmic Reticulum Staining Kit protocol. BBcellProbe E03 or E04 fluorescent dye was first diluted 10-fold from the standard solution, followed by a 20-fold dilution with Hanks’ balanced salt solution (HBSS; MeilunBio, Dalian, China) to prepare the working solution. Cells were washed twice with pre-warmed HBSS, and 1 mL of pre-warmed working solution was added to each well, avoiding light exposure for 30 minutes at 37°C. After staining, cells were fixed with 4% paraformaldehyde for 5 minutes and washed twice with pre-warmed HBSS. Nuclei were counterstained with DAPI for 5 minutes, and slides were mounted with anti-fluorescence quenching sealant. The stained ER structures were visualized using an Olympus FV 1000 laser confocal microscope.

### Protein extraction and enzymatic digestion

HMC3 cells were seeded at a density of 1 × 10^6^ cells per 10 cm dish. Upon reaching 60% confluence, cells were infected with *B. suis* S2 at a MOI of 50 for 2 hours. After infection, the cells from various groups were collected and sonicated on ice for 50 seconds, using 10 second intervals (5 seconds on, 5 seconds off) with a 200 W ultrasonicator. Cellular debris was removed by centrifugation at 14,000 rpm for 5 minutes at 4°C. The resulting protein extracts were quantified using a BCA assay. Reduction of the protein extracts was performed with dithiothreitol (5 mM final concentration) for 30 minutes at 56°C, followed by incubation with iodoacetamide (11 mM final concentration) for 15 minutes at room temperature in the dark. Protein digestion was carried out overnight at 37°C using pancreatin at a 1:50 mass ratio (pancreatin to protein), with an additional 4 hours of digestion at a 1:100 mass ratio.

### Ubiquitinated peptides enrichment

For ubiquitinated peptides enrichment, the tryptic peptides were dissolved in an immunoprecipitation (IP) buffer and centrifuged. The supernatant was incubated with ubiquitination antibody-immobilized resin (lot#: PTM1104; Jingjie PTM Bio Lab, Hangzhou, China) overnight at 4°C on a rotating shaker. Afterward, the resins were washed four times with IP buffer and twice with deionized water. The peptides bound to the resin were eluted using 0.2% trifluoroacetic acid and collected through three rounds of elution. The eluate was then vacuum-dried, and salts were removed. Ubiquitinated peptides were quantified using the label-free quantification (LFQ) approach.

### LC-MS/MS analysis

For liquid chromatography, mobile phase A (0.1% formic acid and 2% acetonitrile) was used to dissolve the peptides, which were then separated on an EASY-nLC 1000 ultra-high-performance liquid chromatography system. The liquid phase gradient was set as follows: 0–40 minutes, 6%–24% mobile phase B (0.1% formic acid and 90% acetonitrile); 40–52 minutes, 24%–38% B; 52–56 minutes, 38%–80% B; 56–60 minutes, 80% B, with a flow rate of 450 nL/min. Following separation, peptides were ionized using NSI ions and analyzed on an Orbitrap Fusion mass spectrometer at an ion source voltage of 2.0 kV. Peptide parent ions and secondary fragments were detected and analyzed using high-resolution Orbitrap, with primary mass spectrometry scans set to a range of 350–1,550 *m*/*z* and a resolution of 60,000. Secondary mass spectrometry scans started at 100 *m*/*z*, with a resolution of 15,000. A data-dependent acquisition (DDA) program was used for data collection, with a threshold ion count set at 5,000 ions/s, a maximum injection time of 200 ms, and an automatic gain control (AGC) target of 5E4. A dynamic exclusion time of 15 seconds was applied during LC-MS/MS data analysis. MaxQuant (v1.5.2.8) was used to obtain secondary mass spectral data. The database used was the Human_SwissProt (20422 sequences) and the FDR for protein or PSM identification was set to 1%.

### Bioinformatic data analysis

To analyze the functions of differentially ubiquitinated proteins, GO annotations were performed using the UniProt-GOA database (http://www.ebi.ac.uk/GOA/). The system converted protein IDs into UniProt IDs, which were subsequently mapped to GO IDs. The ubiquitinated proteins were categorized based on cellular components, molecular functions, or biological processes. The InterPro database (http://www.ebi.ac.uk/interpro/) was then used to annotate the protein domains of the identified ubiquitinated proteins. Further annotation was done through the KAAS service tool (http://www.genome.jp/kaas-bin/kaas_main), and KEGG mapper (http://www.kegg.jp/kegg/mapper.html) was employed to map the annotated proteins to relevant pathways. Subcellular localization analysis was conducted using wolfpsort (http://www.genscript.com/psort/wolf_psort.html). Ubiquitination site motif analysis was performed with MoMo software. GO and KEGG pathway enrichment, along with protein domain enrichment (*P*-value < 0.05), were analyzed using Perl module software (https://metacpan.org/pod/Text::NSP::Measures::2D::Fisher). Heatmaps and clusters were visualized using the heatmap.2 function from the gplots library in R (https://cran.r-project.org/web/packages/cluster/). Protein-protein interaction networks of differentially ubiquitinated proteins (confidence score > 0.7) were analyzed using the STRING database (http://blast.ncbi.nlm.nih.gov/Blast.cgi) and visualized with the R package “networkD3” (https://cran.r-project.org/web/packages/networkD3/).

### Quantitative real-time PCR

HMC3 cell lines were seeded at a density of 4 × 10^5^ cells per 6 cm dish, and *in vitro* bacterial infection experiments were performed when the cells reached 60% confluence. Total RNA was extracted using the RNA simple Total RNA Kit, and reverse transcription of RNA into cDNA was performed with the Transcriptor First-Strand cDNA Synthesis Kit. Primer sequences for Calr and Gapdh were synthesized by Sangon Biotech (Shanghai, China) as follows: *Calr*: Fwd-5′ CCAACGATGAGGCATACGCTGAG 3′, Rev-5′ GCTCCTCGTCCTGTTTGTCCTTC 3′; *Gapdh*: Fwd-5′ CAAGGTCATCCATGACAACTTTG 3′, Rev-5′ GTCCACCACCCTGTTGCTGTAG 3′. The specificity of primers was confirmed via melting curve analysis and agarose gel electrophoresis. Real-time qPCR was conducted using the ChamQ SYBR qPCR Master Mix on a LightCycler 480 II (Roche, Basel, Switzerland). Amplification conditions were set at 95°C for 30 seconds, followed by 40 cycles of 95°C for 10 seconds, 56°C for 30 seconds, and 72°C for 30 seconds, with melting curve analysis at 95°C for 15 seconds, 60°C for 60 seconds, and 95°C for 15 seconds. The melting temperature (Tm) for Calr and Gapdh amplicons was expected to be 84.5°C. Relative Calr mRNA expression was calculated using the 2^−ΔΔCt^ method, with Gapdh serving as the internal control. All RT-qPCR assays were conducted with at least four replicates.

### Flow cytometric analysis

HMC3 cells in each group were digested with pancreatic enzymes (without EDTA) and collected by centrifugation at 800 rpm for 5 minutes. Apoptosis was assessed using the Annexin V-APC/7-AAD Apoptosis Kit, following the manufacturer’s instructions. Briefly, 5 µL of annexin V-APC and 10 µL of 7-AAD staining solution were added to each 100 µL cell suspension, and the mixtures were incubated in the dark at room temperature for 15 minutes. Following incubation, 400 µL of annexin V binding buffer was added to each mixture containing 4 × 10^5^ cells, and apoptosis was quantified using flow cytometry. Single-stained annexin V-APC and 7-AAD cells were used for compensation, and gating was based on control HMC3 cells. Quadrants Q1, Q2, Q3, and Q4 represented necrotic, late apoptotic, early apoptotic, and viable cells, respectively. In this study, the apoptosis rate for each group was calculated as the sum of Q2 and Q3.

### Intracellular calcium levels assessment

For calcium ion assays, HMC3 cells were seeded in 96-well plates at a density of 2 × 10^4^ cells per well. When cells reached 60% confluence, bacterial infection experiments were conducted. The intracellular calcium ion assay was performed using the Intracellular Calcium Staining Kit, as per the instructions. A 500-fold dilution of BBcellProbe F07 fluorescent dye in HBSS was prepared as the working solution, and 100 µL of this solution was added to each well. Cells were incubated at 37°C for 50 minutes in the dark, followed by three washes with pre-warmed HBSS, each for 5 minutes. After the final wash, 100 µL of HBSS was added to each well, and plates were incubated for an additional 30 minutes at 37°C. The fluorescence intensity of intracellular Ca^2+^ was measured using a fluorescence microplate reader (BioTek, Winooski, Vermont, USA).

### Statistical analysis

All experiments were performed with at least three biological replicates, and the data are presented as means ± standard deviations (SD). Statistical analysis was conducted using GraphPad Prism 9.5.1 software. The normality of the data for each group was assessed using the Shapiro-Wilk normality test, and the homogeneity of variances was verified with the Brown-Forsythe test. Statistical differences among groups were determined by one-way analysis of variance (ANOVA), with *P*-values ≤ 0.05 considered statistically significant.

## RESULTS

### Effects of *B. suis* S2 infection on ER stress of HMC3 cells at different times

**Fig 1:**
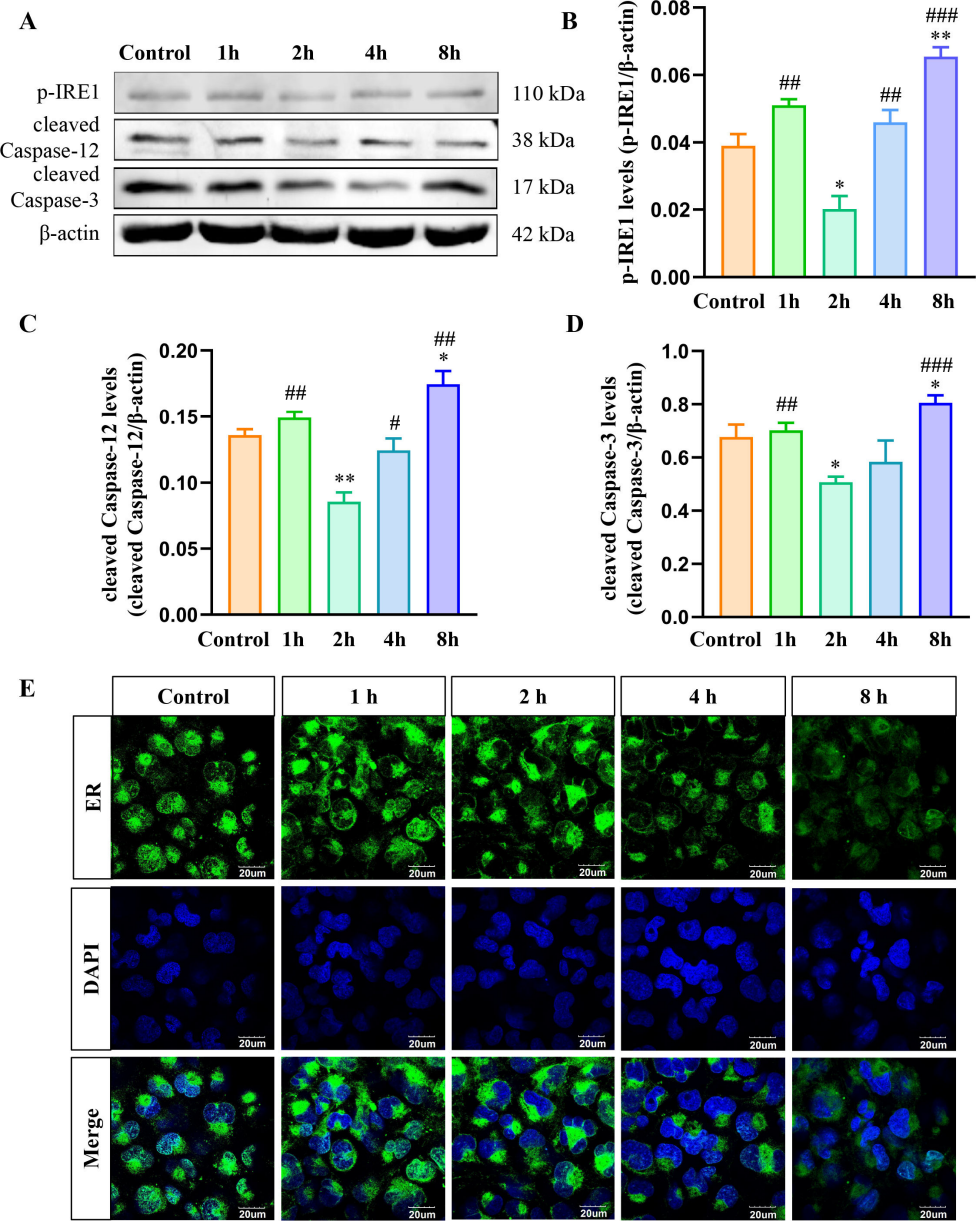
Effects of different *B. suis* S2 infection durations (MOI = 50) on ER and ER stress-related proteins in HMC3 cells. (**A**) Western blot analysis of p-IRE1, cleaved-caspase-12, and cleaved-caspase-3 protein levels in each group. (**B–D**) Quantification of p-IRE1, cleaved caspase-12, and cleaved caspase-3 relative to β-actin. (**E**) ER fluorescence staining using BBcellProbe E03 in each group. Data are from three biologically independent experiments, reported as means ± standard deviations; *P < 0.05, **P < 0.01 versus control; ^#^*P* < 0.05, ^##^*P* < 0.01, ^###^*P* < 0.001 versus 2 hour group.

*B. suis* S2 modulates ER stress in host cells to support its intracellular survival (14). IRE1 plays a pivotal role as the primary receptor in the ER stress response (34), while caspase-12 is a key mediator of ER stress-induced apoptosis (35), both of which become activated during ER stress. Our previous study has verified that *B. suis* S2 could inhibit HMC3 cell apoptosis at an MOI of 50 (36). To explore the impact of *B. suis* S2 infection on ER stress-related apoptosis in HMC3 cells at various time points, the protein levels of p-IRE1, cleaved caspase-12, and cleaved caspase-3 were analyzed using Western blot (Fig. 1A). The results indicated a significant reduction in the levels of p-IRE1, cleaved caspase-12, and cleaved caspase-3 in the 2 hour group compared to the other groups. By contrast, these proteins were significantly elevated in the 8 hour group compared to the control (Fig. 1B through D). To further assess the effect of *B. suis* S2 infection on the ER in HMC3 cells, ER fluorescence staining was performed. The ER fluorescence intensity in 8 hour group was markedly reduced (Fig. 1E). These results suggest that *B. suis* S2 suppresses the IRE1/caspase-12/caspase-3 pathway in HMC3 cells at 2 hours post-infection, while this pathway is activated and ER integrity potentially compromised by 8 hours post-infection.

### Effects of *B. suis* S2 infection on ER stress of HMC3 cells at various MOI

**Fig 2:**
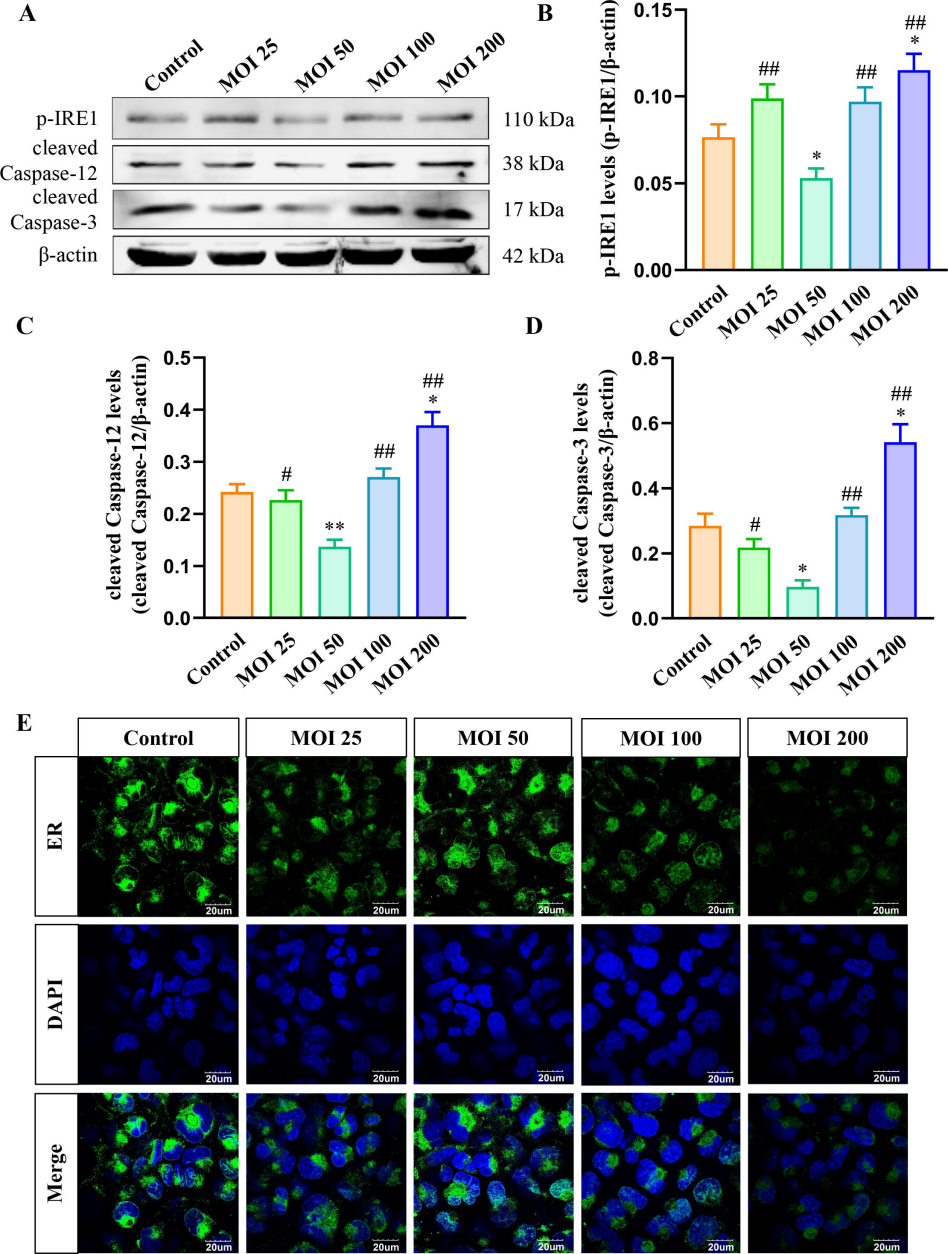
Effects of *B. suis* S2 on ER and ER stress-related proteins in HMC3 cells at different MOIs over 2 hours. (**A**) Western blot analysis of p-IRE1, cleaved-caspase-12, and cleaved-caspase-3 levels in each group. (**B–D**) Quantitative analysis of p-IRE1, cleaved-caspase-12, and cleaved-caspase-3 levels relative to β-actin. (**E**) ER fluorescence staining using BBcellProbe E03 in each group. Data are presented from three independent biological replicates, expressed as means ± standard deviations; *P < 0.05, **P < 0.01 versus control; ^#^*P* < 0.05, ^##^*P* < 0.01 versus MOI 50.

ER stress can lead to apoptosis during prolonged stress (37). By inhibiting ER stress, apoptosis in host cells may be reduced, potentially aiding *Brucella*’s intracellular survival. Thus, this study focused on the inhibitory effects of *B. suis* S2 on ER stress in HMC3 cells and further examined the impact of *B. suis* S2 at varying MOIs for 2 hours. Western blot analysis was performed to assess the levels of p-IRE1, cleaved caspase-12, and cleaved caspase-3 in each MOI group (Fig. 2A). Quantification of these proteins showed that p-IRE1, cleaved caspase-12, and cleaved caspase-3 were significantly reduced in the MOI 50 group compared to the other groups (Fig. 2B through D). Conversely, the levels of these proteins were significantly elevated in the MOI 200 group compared to the control (Fig. 2B through D). In addition, ER fluorescence staining was conducted to evaluate the effects of different MOIs of *B. suis* S2 on the ER. The ER fluorescence intensity in the MOI 200 group was displayed significantly diminished (Fig. 2E). These results indicate that *B. suis* S2 suppresses the IRE1/caspase-12/caspase-3 signaling pathway in HMC3 cells at an MOI of 50 for 2 hours, while the pathway is activated, and ER damage is observed at an MOI of 200.

### LC-MS/MS screening of ubiquitinated proteins

**Fig 3:**
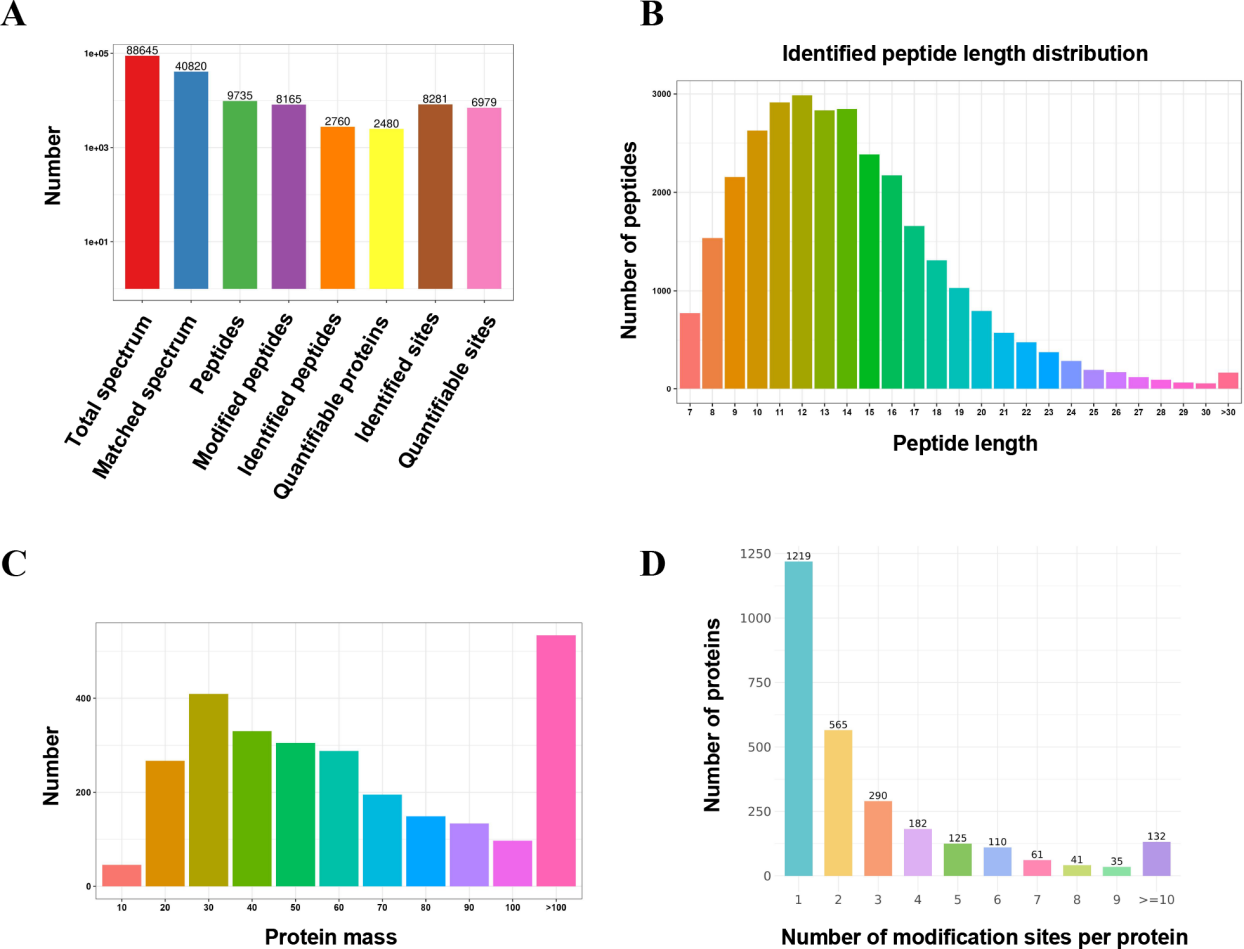
Distribution of peptide length, protein mass, and ubiquitination sites in the identified proteins. (**A**) Overview of the identified peptides and proteins. (**B**) Length distribution of ubiquitinated peptides in *B. suis* S2-infected HMC3 cells. (**C**) Protein mass distribution of ubiquitinated proteins in *B. suis* S2-infected HMC3 cells. (**D**) Distribution of ubiquitination sites in peptides from *B. suis* S2-infected HMC3 cells.

Ubiquitination plays a pivotal role in regulating protein degradation and host innate immune responses (25). Bacterial intracellular survival is significantly influenced by their ability to modulate ubiquitination modifications (38). Building on previous findings, ubiquitination-modified proteins in HMC3 cells infected with *B. suis* S2 at an MOI of 50 for 2 hours were screened using LC-MS/MS analysis. This analysis identified 9,735 peptides, of which 8,165 were ubiquitinated. These peptides corresponded to 2,760 total proteins, with 2,480 proteins being quantified. In addition, 8,281 ubiquitination sites were identified, with 6,979 sites quantified (Fig. 3A). The identified ubiquitinated peptides were further analyzed for length and mass distribution. Peptide lengths ranged from 7 to 35,991 amino acids (aas), with the majority falling between 7 and 22 aas (Fig. 3B). Protein mass distribution ranged from 10 to 100 kDa, with most proteins between 20 and 90 kDa (Fig. 3C). A further investigation into ubiquitination sites revealed that most proteins contained 1 to 3 ubiquitination sites, with 132 proteins exhibiting more than 10 modification sites (Fig. 3D). These results suggest that ubiquitination may play a significant role in the inhibitory effect of *B. suis* S2 on the IRE1/caspase-12/caspase-3 signaling pathway in HMC3 cells.

### Repeatability test and functional classification of differentially ubiquitinated proteins

**Fig 4:**
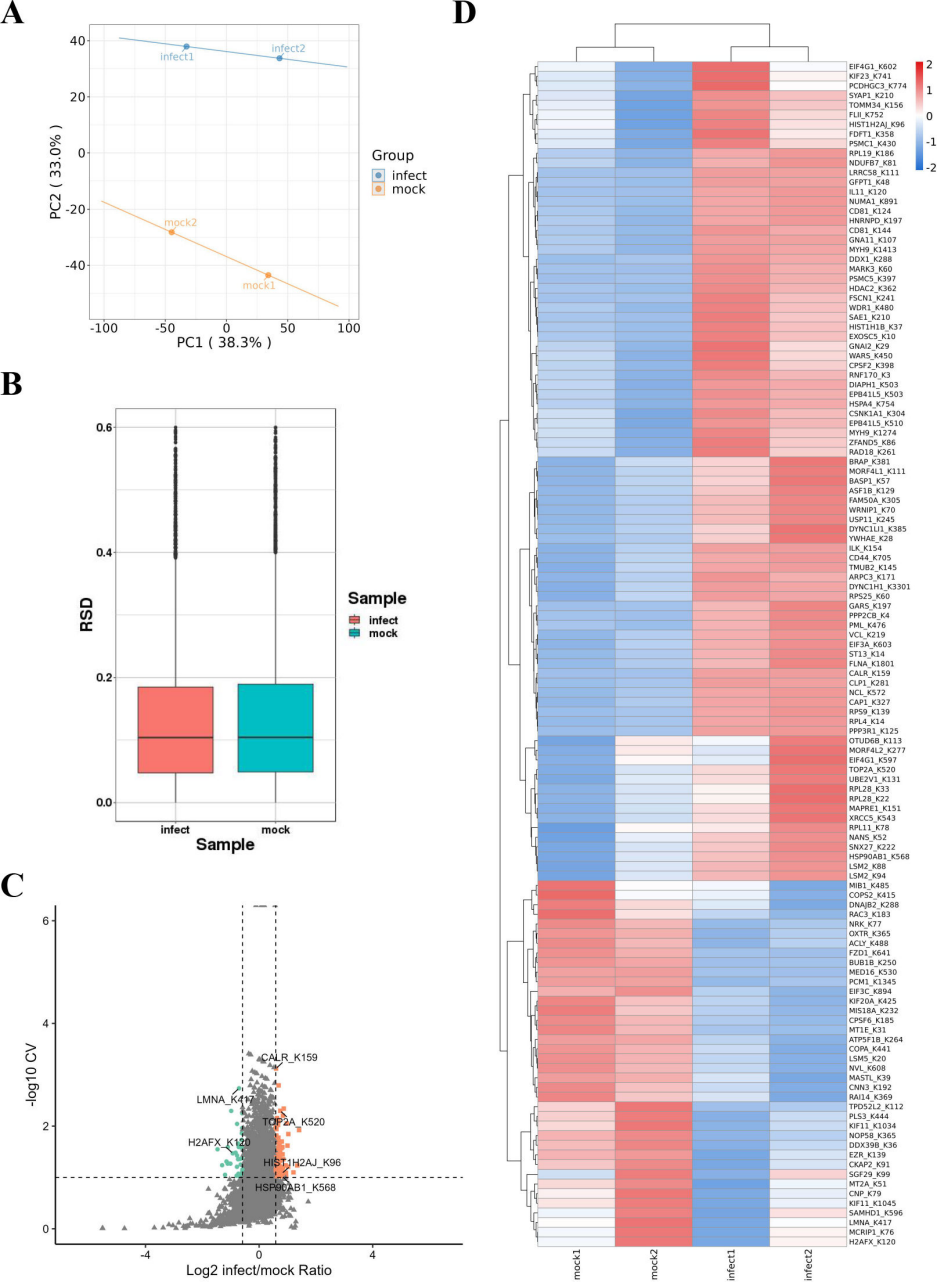
Quality control and quantitative analysis of ubiquitinated proteins. (**A**) PCA plot of differentially ubiquitinated proteins in HMC3 cells post *B. suis* S2 infection. (**B**) Boxplot of RSD for protein quantification in each group, where lower RSD values indicate better repeatability. (**C**) Volcano plot of ubiquitinated proteins in HMC3 cells. Increased ubiquitination is marked in orange-red (CV < 0.1, ratio > 1.5), and decreased in blue-green (CV < 0.1, ratio < 0.67). (**D**) Hierarchical clustering heatmap of differentially ubiquitinated proteins between mock and infection groups.

To assess the consistency and statistical heterogeneity of the quantitative results from biological duplicates, principal component analysis (PCA) and relative standard deviation (RSD) boxplots were employed to evaluate sample repeatability. PCA revealed clear separation between groups and clustering within groups, indicating significant inter-group differences and strong intra-group repeatability (Fig. 4A). The RSD boxplot further confirmed excellent repeatability within each group (Fig. 4B). Based on these analyses, 116 differentially ubiquitinated proteins were identified, with 79 proteins showing increased ubiquitination and 37 proteins exhibiting decreased ubiquitination (Fig. 4C and D; Tables S1 and S2). Cluster analysis confirmed a high reproducibility of samples in both the infected and mock groups and clear differentiation of the differentially ubiquitinated proteins (Fig. 4D). Given that Brucella regulates host cell apoptosis by manipulating ER stress and the unfolded protein response (39), proteins associated with cell death or ER function were prioritized. Six proteins were selected, including four with significantly upregulated ubiquitination and two with notable downregulation (Fig. 4C). These results suggest that *B. suis* S2 infection activates the ubiquitin system in HMC3 cells, potentially implicating numerous ubiquitinated proteins in the regulation of cell fate.

### CALR was screened out by KEGG enrichment and subcellular localization analysis

**Fig 5:**
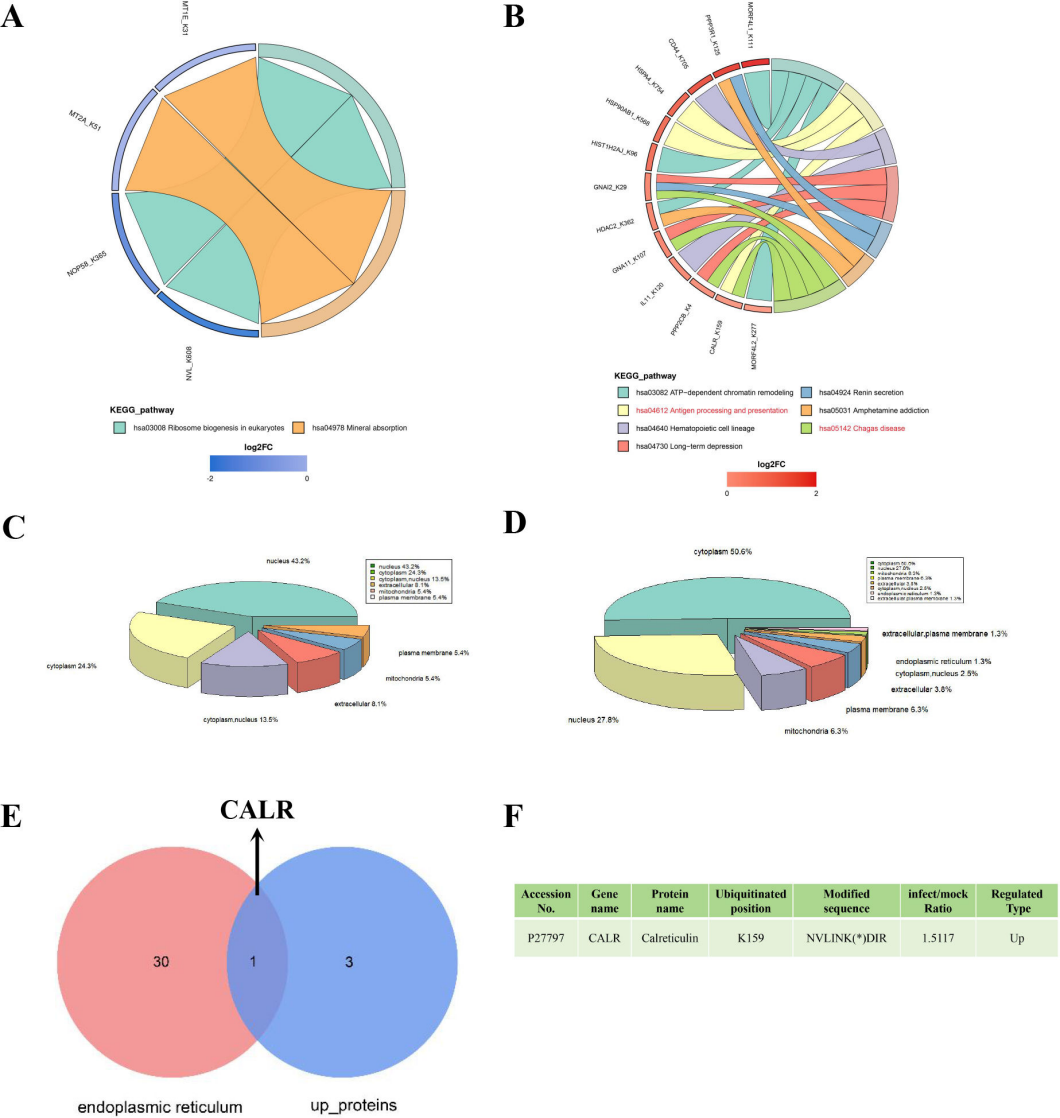
KEGG enrichment and subcellular localization of differentially ubiquitinated proteins in non-infected and *B. suis* S2-infected HMC3 cells. (**A**) Chord diagram showing KEGG enrichment analysis of proteins with significantly decreased ubiquitination. (**B**) Chord diagram for proteins with significantly increased ubiquitination. (**C**) Pie chart depicting the subcellular localization of proteins with decreased ubiquitination. (**D**) Pie chart illustrating the subcellular localization of proteins with increased ubiquitination. (**E**) Venn diagram identifying CALR as an ER-localized protein associated with apoptosis. (**F**) Details of CALR with significantly elevated ubiquitination levels.

To further investigate the functions and pathways associated with the differentially ubiquitinated proteins, KEGG enrichment analysis was performed. Only KEGG pathways with *P*-values below 0.05 were considered, and the results were visualized using chord diagrams. The chord diagram revealed that four proteins with significantly reduced ubiquitination were enriched in pathways related to mineral absorption and ribosome biogenesis in eukaryotes (Fig. 5A). By contrast, 13 proteins with significantly increased ubiquitination were enriched in seven KEGG pathways, including Chagas disease and antigen processing and presentation (Fig. 5B). A literature review revealed that among the differentially ubiquitinated proteins, only CALR was directly linked to apoptosis. Subcellular localization analysis was conducted to identify ER function-related proteins among the differentially ubiquitinated proteins. The results showed that proteins with both decreased and increased ubiquitination were primarily localized in the cytoplasm and nucleus (Fig. 5C and D; Table S3). Notably, 1.3% of the upregulated ubiquitinated proteins were localized in the ER (Fig. 5D; Table S3). To refine the selection of proteins associated with both ER function and cell death, the upregulated ubiquitinated proteins were screened using the keyword “apoptosis,” and CALR was identified as the ER-localized protein among these candidates (Fig. 5D and E; Table S4). In summary, CALR was highlighted as a key ER-associated protein involved in apoptosis.

### The interaction network and validation of CALR expression

**Fig 6:**
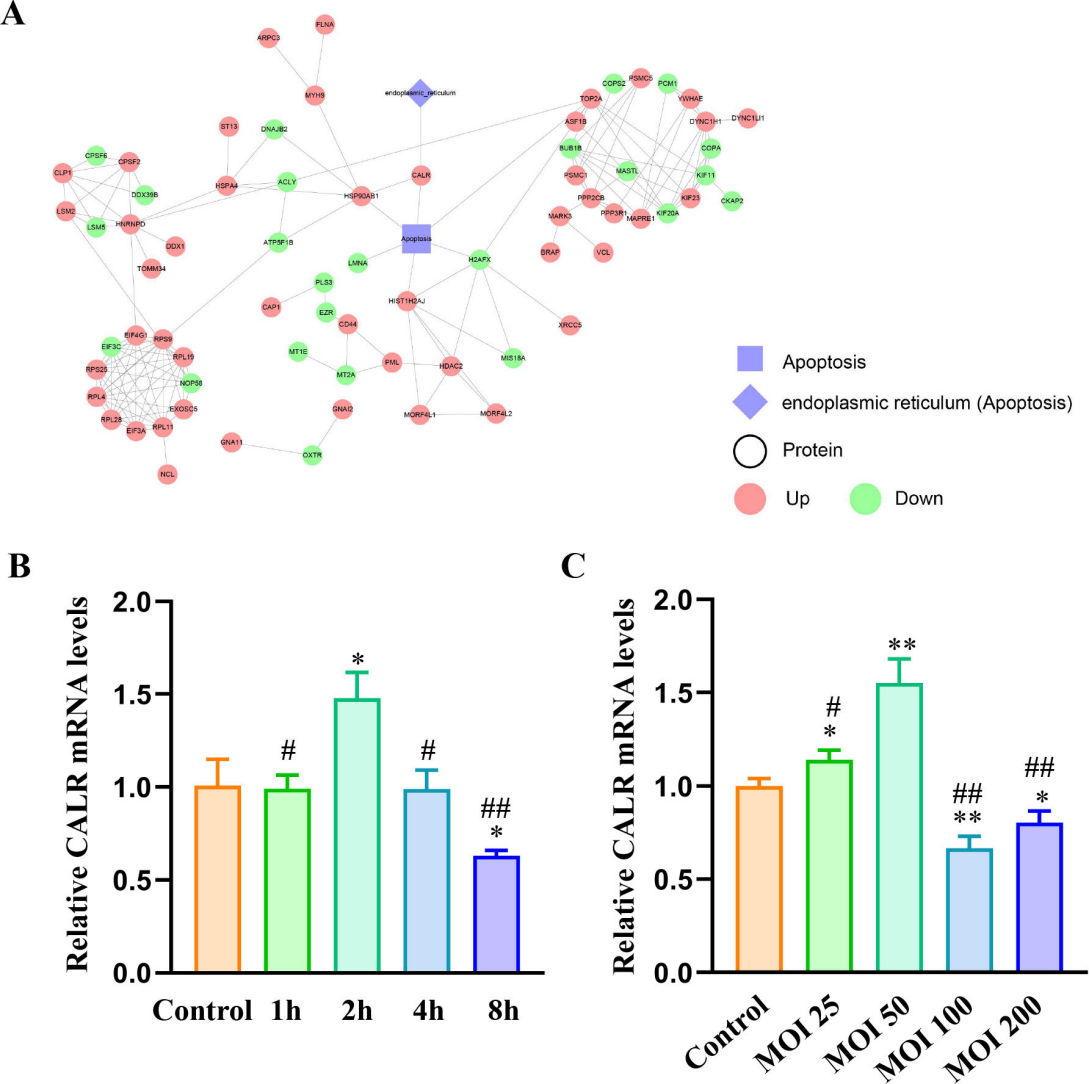
Protein-protein interaction network of CALR and validation of the CALR mRNA levels. (**A**) Interaction network of differentially ubiquitinated proteins associated with apoptosis and ER function. (**B**) Relative expression of CALR mRNA in HMC3 cells infected with *B. suis* S2 (MOI = 50) at different time points. (**C**) CALR mRNA levels 2 hours post-infection with *B. suis* S2 at varying MOIs. Data are presented as means ± standard deviations from three independent biological replicates; *P < 0.05, **P < 0.01 versus control; ^#^*P* < 0.05, ^##^*P* < 0.01 versus 2 h or MOI 50.

To elucidate how other ubiquitinated proteins interact with CALR, a predictive analysis was conducted on differentially ubiquitinated proteins linked to apoptosis and ER function. The analysis identified CALR as the only protein directly associated with ER function (Fig. 6A). Among the apoptosis-related proteins, four (CALR, HSP90AB1, ASF1B, and HIST1H2AJ) showed increased ubiquitination, while two (LMNA and H2AFX) exhibited decreased ubiquitination. Notably, HSP90AB1 was the sole protein found to directly interact with CALR (Fig. 6A; Table S5). Building on these findings, CALR expression was further validated in HMC3 cells infected with *B. suis* S2 at varying MOIs and time points. Previous research confirmed that *B. suis* S2 significantly increased CALR protein levels at an MOI of 50 for 2 hours (36). In this study, HMC3 cells were exposed to *B. suis* S2 at an MOI of 50 for different durations, and CALR mRNA levels were assessed using RT-qPCR. The results indicated a significant elevation of CALR mRNA in the 2 hour group compared to other groups, while a marked decrease was observed in the 8 hour group compared to the control (Fig. 6B). In addition, after 12 hours of infection at varying MOIs, CALR mRNA levels in the MOI 50 group were significantly higher than in the other groups, while levels in the MOI 100 and MOI 200 groups were significantly lower than in the control group (Fig. 6C). Overall, *B. suis* S2 notably increased CALR mRNA levels in HMC3 cells at an MOI of 50 for 2 hours.

### *B. suis* S2 inhibits IRE1/caspase-12/caspase-3 pathway-related apoptosis of HMC3 cells by increasing CALR levels

**Fig 7:**
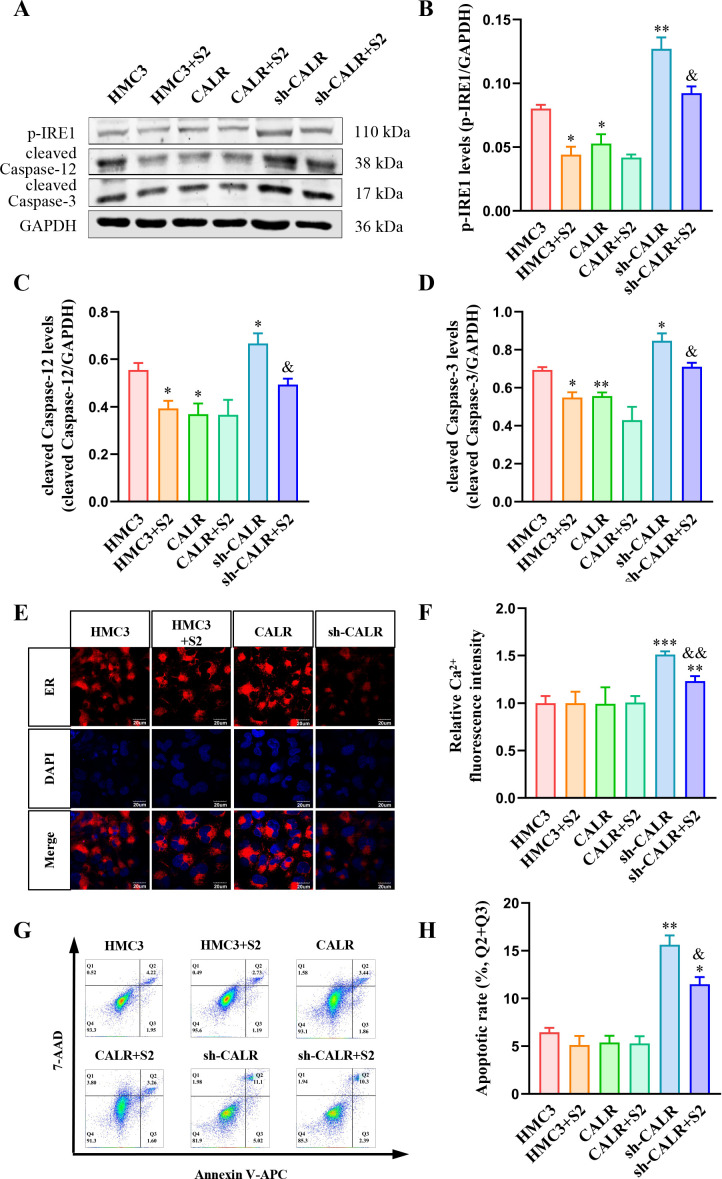
*B. suis* S2 inhibits IRE1/caspase-12/caspase-3 pathway-related apoptosis of HMC3 cells by increasing CALR levels. (**A**) Western blot analysis of p-IRE1, cleaved-caspase-12, and cleaved-caspase-3 protein levels in each group. (**B–D**) Quantitative analysis of p-IRE1, cleaved-caspase-12, and cleaved-caspase-3 levels, normalized to GAPDH. (**E**) Fluorescent staining of the ER using BBcellProbe E04. (**F**) Relative fluorescence intensity of intracellular calcium ions in each group. (**G**) Flow cytometric analysis of apoptosis across groups. (**H**) Apoptotic rate (percentage of cells in Q2 and Q3) across groups. Data are presented as means ± standard deviations from three independent biological replicates; *P < 0.05, **P < 0.01, ***P < 0.001 versus HMC3; ^&^*P* < 0.05, ^&&^*P* < 0.01 versus sh-CALR.

To further explore the relationship between elevated CALR levels and the suppression of the IRE1/caspase-12/caspase-3 pathway following *B. suis* S2 infection in HMC3 cells, HMC3 cell lines, CALR-overexpressing (CALR), and knockdown (sh-CALR) HMC3 cell lines were infected with *B. suis* S2. Previous studies confirmed that *B. suis* S2 infection significantly increased CALR protein levels in all cell lines (39). Western blot analysis was used to assess the protein levels of p-IRE1, cleaved caspase-12, and cleaved caspase-3 (Fig. 7A). Results showed that p-IRE1, cleaved-caspase-12, and cleaved-caspase-3 levels were significantly decreased in the CALR group but markedly increased in the sh-CALR group compared to the control (Fig. 7B through D). This indicates that CALR inhibits the IRE1/caspase-12/caspase-3 pathway. Furthermore, the protein levels were significantly reduced in both the HMC3 + S2 and sh-CALR + S2 groups compared to their corresponding controls (Fig. 7B through D).

Fluorescent staining of the ER was performed to evaluate the effects of *B. suis* S2 infection and CALR on ER function. The results showed reduced ER fluorescence intensity in the sh-CALR group compared to the control, while no significant differences were observed in the HMC3 +S2 and CALR groups (Fig. 7E). This suggests that CALR may protect ER integrity in HMC3 cells. Given that the ER serves as the primary reservoir of intracellular calcium and relies on CALR to sequester free calcium ions (40), intracellular free calcium levels were assessed. Free Ca^2+^ levels were significantly elevated in the sh-CALR and sh-CALR + S2 groups compared to the control, with a marked decrease in the sh-CALR +S2 group compared to sh-CALR alone (Fig. 7F). Flow cytometry was then used to assess apoptosis levels across the different groups (Fig. 7G). Apoptotic rates were significantly higher in the sh-CALR and sh-CALR + S2 groups compared to the control, but the *B. suis* S2-infected sh-CALR group displayed a markedly reduced apoptotic rate compared to the sh-CALR group (Fig. 7H). In summary, CALR maintains ER and cellular homeostasis by suppressing the IRE1/caspase-12/caspase-3 pathway in HMC3 cells. In addition, *B. suis* S2 significantly suppresses apoptosis in HMC3 cells caused by CALR gene knockdown by inhibiting the IRE1/caspase-12/caspase-3 pathway.

## DISCUSSION

Neurobrucellosis is a severe complication of brucellosis caused by the infiltration of *Brucella* spp. into the CNS (41). This condition poses a significant threat to human health and quality of life due to *Brucella*’s ability to persist in microglia, leading to chronic infections (8). As the resident macrophages of the CNS, microglia play critical roles in clearing dead neurons and eliminating pathogens (42). Once *Brucella* invades and is engulfed by macrophages, it forms BCVs, which undergo remodeling by the ER, facilitating bacterial replication (43, 44). *Brucella* then manipulates ER stress to suppress host cell apoptosis, supporting its intracellular survival and promoting chronic infection and inflammation (45). However, the precise molecular mechanisms by which *Brucella* modulates apoptosis through ER stress regulation remain incompletely understood.

The ER plays a vital role in protein synthesis and transport (46). Infections or inflammation can disrupt protein folding, leading to the accumulation of misfolded proteins and the induction of ER stress (47). ER stress sensors are pivotal in this process, with IRE1 being the most conserved and extensively studied (48). IRE1 is critical in determining the fate of host cells during intracellular pathogen infection (49, 50). Studies have shown that *Brucella abortus* or *Brucella melitensis* induces ER stress by activating the IRE1 pathway in macrophages (51). Conversely, other research suggests that *Brucella abortus* can attenuate ER stress in trophoblast cells by inhibiting IRE1 activation (18). Our findings demonstrated that *B. suis* S2 reduces p-IRE1 levels in HMC3 cells at an MOI of 50 for 2 hours, but activates IRE1 at an MOI of 200 or after 8 hours of infection. This suggests that *Brucella*’s modulation of IRE1 is dependent on factors such as infection duration, MOI, bacterial strain, and host cell type. Given our focus on understanding how *Brucella* establishes chronic infections, the inhibitory effects of *B. suis* S2 on ER stress were examined.

Caspase-12, specifically localized to the ER, plays a critical role in apoptosis triggered by ER stress (52). Once activated (cleaved caspase-12), it initiates caspase-3, leading to apoptosis (53). The findings demonstrated that *B. suis* S2 not only reduced p-IRE1 levels but also lowered cleaved caspase-12 and cleaved caspase-3 protein levels, indicating that *B. suis* S2 alleviates ER stress by inhibiting the IRE1/caspase-12/caspase-3 pathway. Previous research by Wu et al. confirmed that this pathway induces ER stress in pancreatic acinar cells (54). Upon ER stress induction, a marked decrease in ER fluorescence intensity is observed when stained with fluorescent dyes (55). This aligns with the results, showing that *B. suis* S2 activates the IRE1/caspase-12/caspase-3 pathway and reduces ER fluorescence intensity at an MOI of 200 or after 8 hours of infection. However, at an MOI of 50 for 2 hours, *B. suis* S2 suppressed this pathway without affecting ER fluorescence intensity. Thus, the focus shifted to the inhibitory effect of *B. suis* S2 on the IRE1/caspase-12/caspase-3 signaling pathway.

Considering these findings, it is speculated that *B. suis* S2 may regulate ER stress in HMC3 cells by modulating ubiquitination, as this process often leads to enhanced proteolytic degradation and a subsequent reduction in protein levels (56). Ubiquitination is one of the most widespread and critical post-translational modifications (27). Through this process, proteins are covalently tagged with ubiquitin via ubiquitin ligase, followed by degradation by proteolytic enzymes, ensuring cellular protein homeostasis (57). Pathogens often exploit the host’s ubiquitination machinery to manipulate cellular processes for their survival (38). For instance, Xia et al. demonstrated that *Mycobacterium tuberculosis* promotes the ubiquitination of HIF-1α in macrophages to evade immune responses and enhance survival (58). Similarly, *Brucella*, an intracellular bacterium, has been shown to regulate ubiquitination in host cells. Identifying ubiquitinated proteins may offer insights into how *B. suis* S2 inhibits the IRE1/caspase-12/caspase-3 pathway. LC-MS/MS-based ubiquitin-modified proteomics have proven effective in studying pathogen-host interactions (59). Thus, this study employed proteomics technology to identify differentially ubiquitinated proteins in *B. suis* S2-infected HMC3 cells, aiming to elucidate the link between ubiquitination and the IRE1/caspase-12/caspase-3 pathway.

In this study, 37 downregulated and 79 upregulated ubiquitinated proteins were identified (Fig. S1). KEGG enrichment analysis revealed that downregulated ubiquitinated proteins were primarily enriched in the hsa04978 and hsa03008 pathways, while upregulated ubiquitinated proteins were significantly involved in seven pathways (hsa05142, hsa04612, hsa04640, hsa04730, hsa03082, hsa04924, and hsa05031). Among these, the focus was placed on signaling pathways related to pathogen infection. Consequently, two pathways (hsa05142 and hsa04612) were selected for further analysis. The GNAI2, CALR, GNA11, and PPP2CB proteins were enriched in the hsa05142 pathway, while HSP90AB1, CALR, and HSPA4 were mapped to the hsa04612 pathway. Notably, CALR was enriched in both pathways. Cockram et al. found that CALR is transported to the surface of LPS-activated microglia to enhance bacterial binding and phagocytosis (60). In addition, CALR is involved in immune responses, as shown by Santara et al., who reported that NK cells recognize surface CALR on Zika virus-infected JEG-3 cells (61). GNAI2 also plays a role in immune responses, with Álvarez et al. demonstrating its differential expression in Ibizan hounds following *Leishmania infantum* infection (62). HSP90AB1, another key protein in host resistance to pathogens, has been shown to facilitate viral survival by regulating autophagy in Classical Swine Fever Virus-infected PK-15 cells (63). These studies support our findings that the identified proteins are closely linked to pathogen infection. However, unlike previous research, this study emphasizes the interactions between *B. suis* S2 and microglia.

*Brucella* promotes its replication by forming BCVs, which then fuse with the ER in host cells (64) and ensure long-term survival by regulating host cell apoptosis (32). To explore this further, the subcellular localization of differentially ubiquitinated proteins was analyzed, focusing specifically on those localized to the ER. Among these proteins, only CALR was found to be linked to apoptotic functions. CALR, a chaperone protein primarily located in the ER lumen (65), plays a role in protein quality control (66) as well as apoptosis regulation (67). Several studies have highlighted the anti-apoptotic properties of CALR. For instance, Sun et al. reported that the cardioprotective effect of adiponectin was mediated by CALR’s anti-apoptotic function (68), while Jiao et al. found that CALR overexpression inhibited fibroblast-like synoviocyte apoptosis in rheumatoid arthritis (69). To gain further insight, an ER- and apoptosis-related protein-protein interaction (PPI) network was constructed. The analysis revealed that CALR was the only protein associated with ER function, while several proteins, including HSP90AB1, TOP2A, HIST1H2AJ, LMNA, and H2AFX, were linked to apoptosis. Studies have confirmed the roles of these proteins in apoptosis regulation. For example, Peng et al. demonstrated that HSP90AB1 interacts with Bcr-Abl to inhibit its activation, thereby inducing apoptosis in chronic myeloid leukemia cells (70). Duan et al. reported that downregulation of TOP2A activated the FOXO signaling pathway, leading to apoptosis in trophoblast cells (71). In addition, Wu et al. showed that the loss of the LMNA gene in mice resulted in cardiac cell apoptosis (72). These results align with and support the results of the PPI analysis, confirming the roles of these proteins in apoptosis regulation.

This study examined the increased ubiquitination of CALR protein. Although theoretically, ubiquitination should lead to a decrease in CALR levels, previous findings demonstrated that *B. suis* S2 significantly elevated CALR protein levels in HMC3 cells at an MOI of 50 for 2 hours (39). This suggests a compensatory upregulation of CALR expression in response to its ubiquitination-induced degradation, as indicated by RT-qPCR results showing a significant rise in CALR mRNA levels under these conditions. The findings align with research by Wang et al., who reported that the intracellular pathogen *Edwardsiella tarda* similarly increased CALR mRNA and protein levels in black rockfish to combat infection (73). Current literature presents contrasting views on CALR’s role in ER stress. Wang et al. found that arsenite-induced ER stress in HT-22 cells activated the calpain2/caspase-12 pathway by promoting CALR expression (74), whereas Tsai et al. demonstrated that miR-92a-1-5p triggered ER stress and kidney injury in mice by inhibiting CALR expression (75). In line with Tsai’s findings, this study confirmed that *B. suis* S2 infection and CALR overexpression suppressed IRE1/caspase-12/caspase-3 pathway-mediated ER stress in HMC3 cells, while CALR knockdown had the opposite effect. Furthermore, as marker of ER stress, CALR is associated with enhanced immune response in breast cancer (76) and systemic lupus erythematosus (77). Taken together, these results suggest that CALR’s involvement in ER stress regulation is context-dependent, varying according to exposure conditions, spectrum of diseases, and cell types.

In addition to its immune-related functions, CALR plays key roles in maintaining ER homeostasis, binding free calcium (39), and regulating apoptosis (78). Our findings confirmed that CALR knockdown impaired ER function and increased intracellular calcium concentration in HMC3 cells. This can be attributed to reduced Ca^2+^-binding capacity within the ER due to diminished CALR levels, which subsequently led to elevated cytosolic free calcium (79). Interestingly, *B. suis* S2 infection did not affect ER integrity or intracellular calcium levels in HMC3 cells. The role of CALR in ER stress-associated apoptosis remains a topic of debate. Our study indicated that CALR knockdown significantly induced apoptosis in HMC3 cells, whereas *B. suis* S2 infection and CALR overexpression did not influence apoptotic activity. Notably, *B. suis* S2 inhibited apoptosis triggered by CALR knockdown in HMC3 cells by suppressing the IRE1/caspase-12/caspase-3 pathway. This finding contrasts with the results of Taguchi et al., who reported that Brucella induced apoptosis through activation of IRE1-mediated ER stress (79). However, Zhi et al. similarly found that *Brucella* suppressed ER stress-associated apoptosis by inhibiting the IRE1 pathway to promote intracellular survival (80), which aligns with our result. The discrepancies between these studies could stem from differences in Brucella species, cell types used, or variations in experimental conditions. Certainly, our finding has some limitations in that we only focused on the regulation of CALR to the ER stress-related apoptosis and only confirmed that CALR could regulate apoptosis via the IRE1/caspase-12/caspase-3 pathway. In addition to the IRE1 axis, the ER stress sensors also include ATF6 and PERK (81). CALR may also exert apoptotic regulatory effects through them, which will be the contents of our subsequent study.

In conclusion, this study confirms that *B. suis* S2 inhibits apoptosis in HMC3 cells by modulating the IRE1/caspase-12/caspase-3 pathway through CALR ubiquitination. A major limitation of this research is the lack of *in vivo* validation, which will be essential in future studies. In addition, identifying the specific ubiquitination sites on CALR targeted by *B. suis* S2 will be crucial. Although *in vitro* experiments could not fully replicate the *in vivo* environment, our experiments simulated phagocytosis and the changes in ER stress-associated protein levels of microglia during *B. suis* S2 invasion, which provides the molecular and cellular basis for further *in vivo* research. Overall, these findings provide a theoretical foundation for understanding the mechanisms underlying neurobrucellosis. Especially the changes in the levels of CALR and its regulated ER stress-related proteins, which provide new insights for us to further screen for effective drugs that interact with them. These will provide the basis for the pharmacological treatments of neurobrucellosis.

## Data Availability

The original data supporting the findings of this study are available in the article, its supplemental material, and relevant online repositories. The mass spectrometry proteomics data have been submitted to the ProteomeXchange Consortium through the PRIDE partner repository, under the data set identifier PXD056006 (http://www.proteomexchange.org/). For additional information, inquiries can be directed to the corresponding authors.

## SUPPLEMENTAL MATERIAL

Fig. S1 msphere.00941-24-s0001.pdf The statistical figure of differentially ubiquitinated proteins and sites.

Fig. S2 msphere.00941-24-s0002.pdf Biological process enrichment analysis of differentially ubiquitinated proteins.

Legends msphere.00941-24-s0003.doc Supplemental material legends.

Table S1 msphere.00941-24-s0004.xlsx KEGG enrichment information of all proteins with reduced ubiquitination modification.

Table S2 msphere.00941-24-s0005.xlsx KEGG enrichment information of all proteins with increased ubiquitination modification.

Table S3 msphere.00941-24-s0006.xlsx Subcellular classification of differentially ubiquitinized proteins.

Table S4 msphere.00941-24-s0007.xlsx Venn overlap data on CALR protein.

Table S5 msphere.00941-24-s0008.xlsx Protein-protein interaction network of CALR.

ASM does not own the copyrights to Supplemental Material that may be linked to, or accessed through, an article. The authors have granted ASM a non-exclusive, world-wide license to publish the Supplemental Material files. Please contact the corresponding author directly for reuse.
