# Supplementary material for: Brucella suis S2 strain inhibits IRE1/caspase-12/caspase-3 pathway-mediated apoptosis of microglia HMC3 by affecting the ubiquitination of CALR
Source: mSphere. 2025 Feb 28;10(3):e00941-24. doi: 10.1128/msphere.00941-24 (PMC11934333; doi:10.1128/msphere.00941-24)
Supplement: Fig. S1 — The statistical figure of differentially ubiquitinated proteins and sites. [file msphere.00941-24-s0001.pdf]

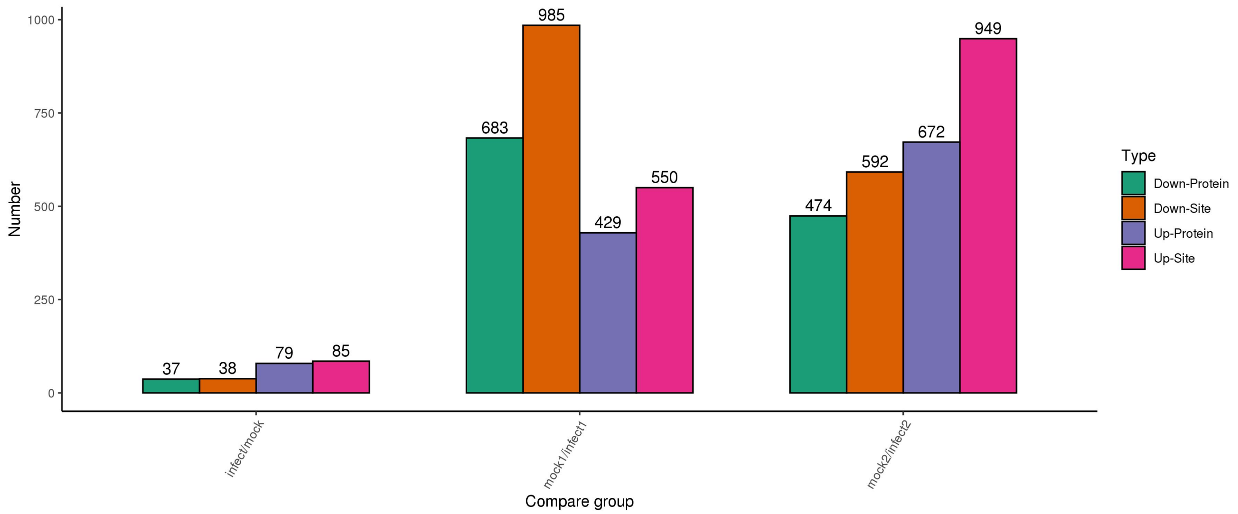

Supplementary Figure 1. The statistical figure of differentially ubiquitinated proteins and sites. HMC3 cells were infected with *B. suis* S2 at an MOI of 50 for 2 hours, the differentially ubiquitinated proteins and sites were identified with modified proteomic technology.
