# Supplementary material for: Brucella suis S2 strain inhibits IRE1/caspase-12/caspase-3 pathway-mediated apoptosis of microglia HMC3 by affecting the ubiquitination of CALR
Source: mSphere. 2025 Feb 28;10(3):e00941-24. doi: 10.1128/msphere.00941-24 (PMC11934333; doi:10.1128/msphere.00941-24)
Supplement: Fig. S2 — Biological process enrichment analysis of differentially ubiquitinated proteins. [file msphere.00941-24-s0002.pdf]

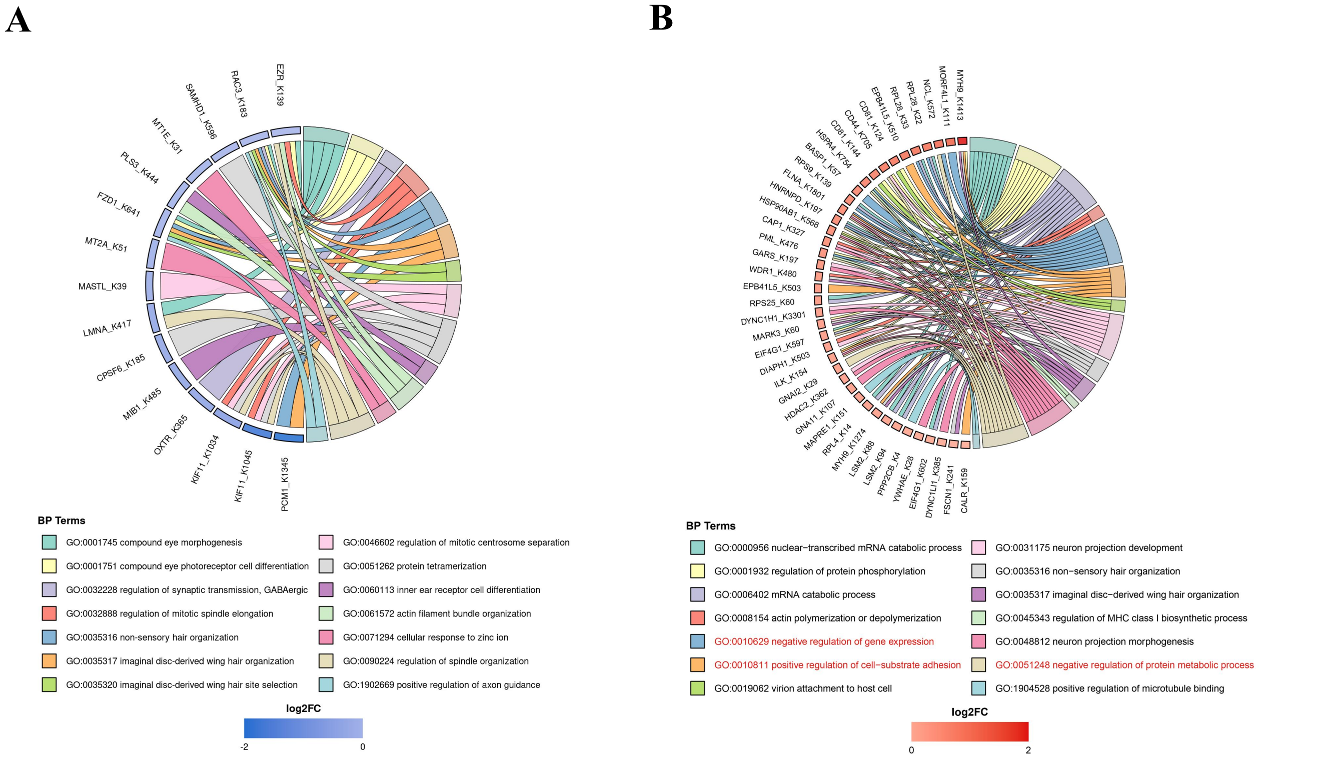

Supplementary Figure 2. Biological process enrichment analysis of differentially ubiquitinated proteins. **(A)** Chord diagram showing biological process enrichment analysis of proteins with significantly decreased ubiquitination. **(B)** Chord diagram showing KEGG enrichment analysis of proteins with distinctly increased ubiquitination.
