# Supplementary material for: Brucella suis S2 strain inhibits IRE1/caspase-12/caspase-3 pathway-mediated apoptosis of microglia HMC3 by affecting the ubiquitination of CALR
Source: mSphere. 2025 Feb 28;10(3):e00941-24. doi: 10.1128/msphere.00941-24 (PMC11934333; doi:10.1128/msphere.00941-24)
Supplement: Legends — Supplemental material legends. [file msphere.00941-24-s0003.doc]

Supplemental Material Legends

Fig. S1: The statistical figure of differentially ubiquitinated proteins and sites.

Fig. S2: Biological process enrichment analysis of differentially ubiquitinated proteins.

Table S1: KEGG enrichment information of all proteins with reduced ubiquitination modification.

Table S2: KEGG enrichment information of all proteins with increased ubiquitination modification.

Table S3: Subcellular classifying of differentially ubiquitinized proteins.

Table S4: Venn overlap data on CALR protein.

Table S5: Protein-protein interaction network of CALR
